# A Web-Based, Computer-Tailored Smoking Prevention Program to Prevent Children From Starting to Smoke After Transferring to Secondary School: Randomized Controlled Trial

**DOI:** 10.2196/jmir.3794

**Published:** 2015-03-09

**Authors:** Henricus-Paul Cremers, Liesbeth Mercken, Math Candel, Hein de Vries, Anke Oenema

**Affiliations:** ^1^Maastricht UniversityDepartment of Health PromotionSchool for Public Health and Primary Care (CAPHRI)MaastrichtNetherlands; ^2^Maastricht UniversityDepartment of Methodology and StatisticsSchool for Public Health and Primary Care (CAPHRI)MaastrichtNetherlands

**Keywords:** Web-based intervention, primary school children, smoking prevention, prompts, computer-tailoring

## Abstract

**Background:**

Smoking prevalence rates among Dutch children increase rapidly after they transit to secondary school, in particular among children with a low socioeconomic status (SES). Web-based, computer-tailored programs supplemented with prompt messages may be able to empower children to prevent them from starting to smoke when they transit to secondary school.

**Objective:**

The main aim of this study is to evaluate whether computer-tailored feedback messages, with and without prompt messages, are effective in decreasing children’s smoking intentions and smoking behavior after 12 and 25 months of follow-up.

**Methods:**

Data were gathered at baseline (T0), and after 12 months (T1) and 25 months (T2) of follow-up of a smoking prevention intervention program called Fun without Smokes. A total of 162 schools were randomly allocated to a no-intervention control group, an intervention prompt group, or an intervention no-prompt group. A total of 3213 children aged 10 to 12 years old participated in the study and completed a Web-based questionnaire assessing their smoking intention, smoking behavior, and sociocognitive factors, such as attitude, social influence, and self-efficacy, related to smoking. After completion, children in the intervention groups received computer-tailored feedback messages in their own email inbox and those messages could be accessed on the intervention website. Children in the prompt group received prompt messages, via email and short message service (SMS) text messaging, to stimulate them to reuse the intervention website with nonsmoking content. Multilevel logistic regression analyses were performed using multiple imputations to assess the program effects on smoking intention and smoking behavior at T1 and T2.

**Results:**

A total of 3213 children participated in the Fun without Smokes study at T0. Between T0 and T1 a total of 1067 children out of the original 3213 (33.21%) dropped out of the study. Between T0 and T2 the number of children that did not participate in the final measurement was 1730 out of the original 3213 (53.84%). No significant program effects were observed for any of the intervention groups compared to the control group at T1 for the intention to engage in smoking—prompt, OR 0.67 (95% CI 0.30-1.50), no-prompt, OR 0.76 (95% CI 0.34-1.67)—or for smoking behavior—prompt, OR 1.13 (95% CI 0.13-9.98), no-prompt, OR 0.50 (95% CI 0.04-5.59). Similar nonsignificant program effects were found at T2 for the intention to start smoking—prompt, OR 0.78 (95% CI 0.26-2.32), no-prompt, OR 1.31 (95% CI 0.45-3.82)—and smoking behavior—prompt, OR 0.53 (95% CI 0.12-2.47), no-prompt, OR 1.01 (95% CI 0.24-4.21).

**Conclusions:**

This study showed that the Web-based, computer-tailored feedback messages with and without prompt messages were not effective in modifying children’s smoking intentions and smoking behavior as compared to no information. Future smoking prevention interventions are recommended to start closer to the age of actual smoking uptake. Furthermore, future studies on Web-based, computer-tailored smoking prevention programs should focus on assessing and controlling exposure to the educational content and the response to the prompt messages.

**Trial Registration:**

Netherlands Trial Register NTR3116; http://www.trialregister.nl/trialreg/admin/rctview.asp?TC=3116 (Archived by WebCite at http://www.webcitation.org/6O0wQYuPI).

## Introduction

Smoking among children and adolescents remains a public health problem [1-3], potentially leading to chronic diseases, cardiovascular diseases, or cancer at a later age [4,5]. Although smoking prevalence among Dutch primary school children at the age of 12 has decreased in the past decade from a range of 2% to 5%, down to 0% [6,7], prevalence still increases rapidly when children are in secondary school (15% of children are monthly smokers at age 14) [6]. One prevention strategy suggests starting smoking prevention programs at primary school, before positive beliefs toward smoking are formed [8]. Given the advantages of a Web-based computer-tailored approach (ie, reduced cost and an expanded reach of participants) [9-11] and the increasing use of the Internet among Dutch children (ie, 96% of 11- to 14-year-olds use the Internet) [12], Web-based computer-tailored smoking prevention programs may be helpful in decreasing smoking initiation rates among children.

Prior research already indicated that schools are an effective setting for reaching children and promoting a healthy lifestyle [13,14]. Hence, numerous smoking prevention programs have been developed to prevent the uptake or continuation of smoking among children and adolescents in school settings [15,16]. Most of the foregoing prevention programs were in-school interventions, however, schools are known to have limited time [14] and teachers are often not educated to perform health promotion activities [13,17]. Therefore, out-of-school interventions may be promising. The Octopus intervention, developed by Ausems et al, is an out-of-school, computer-tailored smoking prevention program (ie, students completed the questionnaire at school and received computer-tailored feedback letters at home sent by postal mail) for Dutch primary school children, aged 11 to 12 years. This program was reported to be more effective as compared to an in-school or combined (ie, in-school and out-of-school) program after 6 months of follow-up [18]. However, this program was provided via postal letters and did not use digital delivery channels, such as the Internet. Previous studies [9,10,19] reported Web-based, computer-tailored programs to be effective in changing unhealthy behaviors among adults, adolescents, and children. Positive program effects have also been observed in Web-based, computer-tailored smoking prevention and cessation studies among adolescents [20,21]. However, despite the positive findings of a pilot study [22], no conclusive evidence is currently available concerning the efficacy of a Web-based, computer-tailored smoking prevention program for primary school children. In this study, the paper-based Octopus intervention was translated to a Web-based, computer-tailored smoking prevention intervention and evaluated for effectiveness among children in the final grades of primary school and after their transition to secondary school. Although Web-based interventions have numerous benefits, a disadvantage is the consistently low reported adherence rates of participants [23,24]. This requires the use of optimal strategies to improve adherence. According to previous research [25-29] prompt messages may be effective in stimulating participants to reuse a Web-based intervention. However, using prompt messages in smoking prevention trials has not been studied among children before.

The aim of this study was to evaluate whether computer-tailored feedback messages, with and without prompt messages, are effective in decreasing the smoking intentions and smoking behavior of Dutch primary school children, aged 10 to 12 years, after 12 and 25 months of follow-up. Furthermore, it is known that children with a low socioeconomic status (SES) engage in smoking more often [30] and have a higher intention to start smoking [31], as compared to high SES children. Therefore, we will also assess whether SES moderates the effects of the two versions of the intervention.

## Methods

### Study Design, Participants, and Procedure

**Figure 1:**
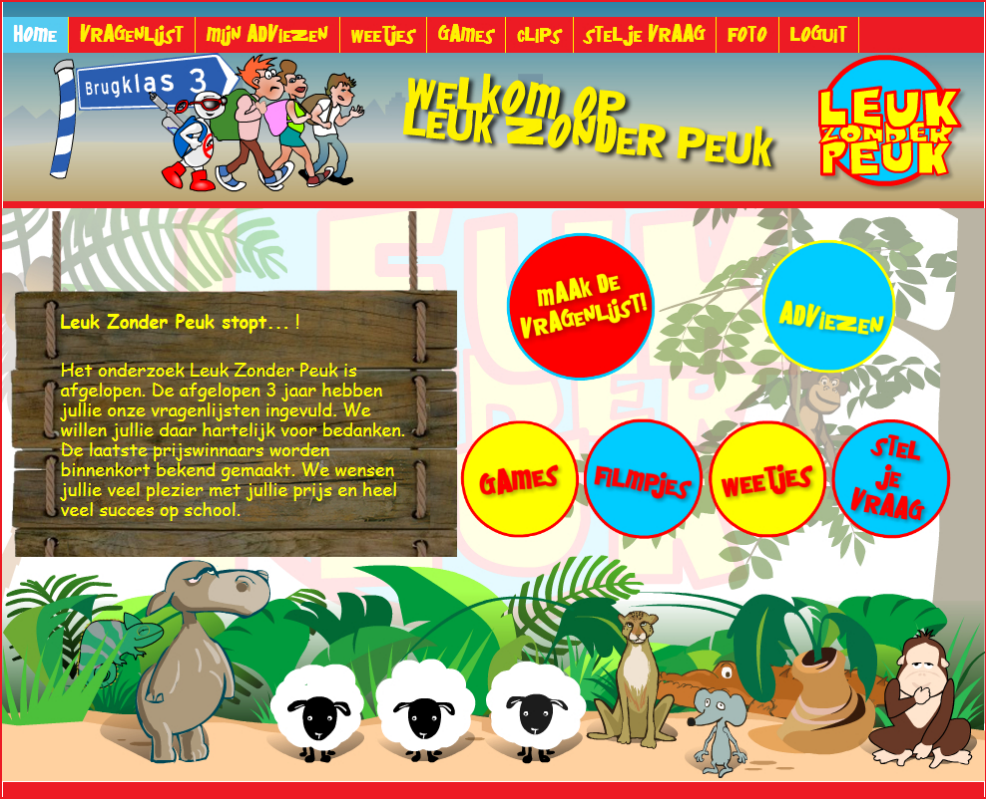
Homepage of the Fun without Smokes website.

A cluster randomized controlled trial with three study arms was conducted to evaluate the effects of the Web-based, computer-tailored smoking prevention intervention, Fun without Smokes [32](Figure 1), on the intention to start smoking and smoking behavior. Children were followed for 2 years. During this period, children participated at three measurement sessions: baseline (T0, October to November 2011), follow-up at 12 months (T1, October to November 2012) and follow-up at 25 months (T2, November to December 2013). Primary schools, as the unit of randomization, were randomly assigned to one of the three study arms (ie, prompt, no-prompt, or control group) in a computer-determined sequence using a clustered randomization scheme.

The Fun without Smokes study was approved by the Medical Ethics Committee of the Atrium-Orbis-Zuyd Hospital (NL32093.096.11 / MEC 11-T-25) and registered in the Netherlands Trial Register (NTR3116).

**Figure 2:**
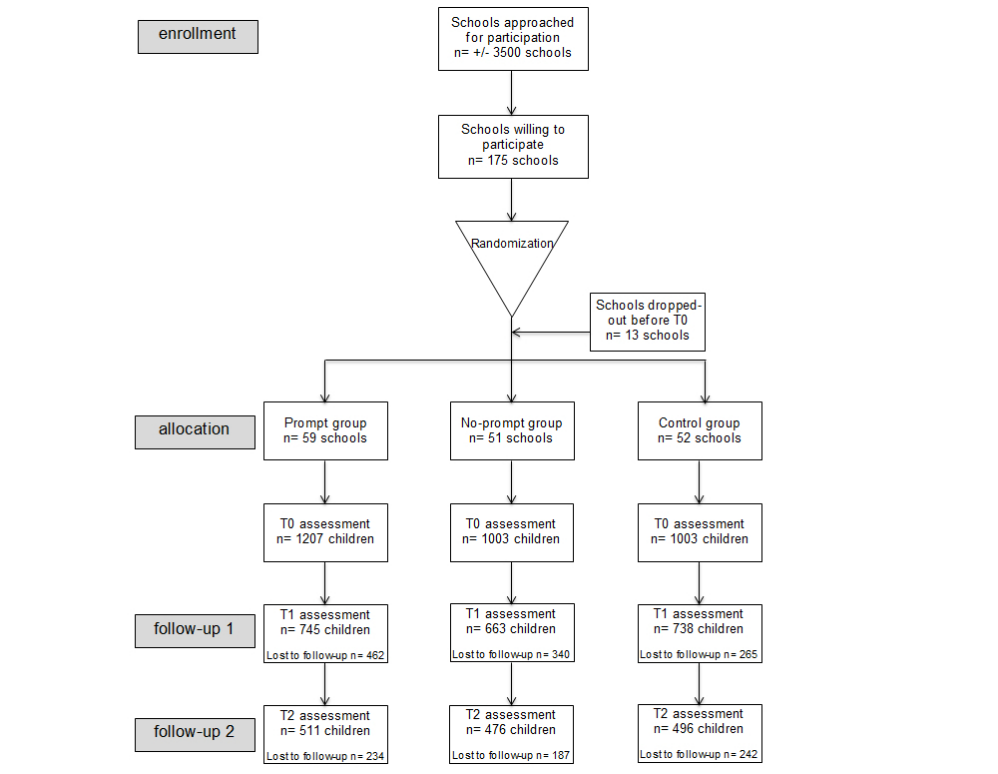
Fun without Smokes study flowchart of baseline (T0), 12-month follow-up (T1), and 25-month follow-up (T2) measurement sessions.

Based on a sample size calculation, 81 schools and 3240 children were needed at T0 in the Fun without Smokes study. This calculation predicted that 15% of the no-prompt group, 8% of the prompt group, and 24% of the control group would have smoked by T2. Since children had to complete the final Web-based questionnaire outside of school, it was taken into account that 60% of the participating children at T0 would have dropped out by T2. The Optimal Design program of Raudenbush [33] was used to calculate the sample size (ie, two-sided testing with Type I error rate=.05, power=.80, intraclass correlation=.04). For the present study, approximately 3500 primary schools were approached by seven Dutch Municipal Health Promotion Organizations and Maastricht University. A total of 162 primary schools participated at T0 in the intervention study with 3213 children. In Figure 2 **,** a flowchart shows the number of participating children and schools at T0, T1, and T2. Children of all participating schools were included in the intervention trial at T0 unless they or their parents refused to be involved—of all participants, 1.7% refused to be involved at T0. The participating children in this study at T0 were Dutch primary school children in grade 7, aged 10 to 11 years. The students were followed when they entered grade 8 (T1) and when they transferred to secondary school (T2). Children had to complete a Web-based questionnaire concerning their smoking behavior, smoking intention, and sociocognitive factors related to smoking (ie, attitude, social influence, and self-efficacy expectations). After completion of the questionnaire, children in the prompt and no-prompt group received computer-tailored feedback messages via email and at the Fun without Smokes website.

At T0 and T1, children completed the Web-based questionnaire at their primary school under supervision by their teacher. At T2, children made the transition to secondary school and had to complete the Web-based questionnaire outside of school on their own initiative. During this period, all children who participated at T0 received an information letter sent by postal mail to their home address asking them to complete the Web-based questionnaire for the last time. In this information letter, children were also informed that they could win one of 500 incentives (eg, film voucher, gift card, or a subscription to a magazine) that would be raffled off among the children that filled out the final questionnaire completely. If children had provided their email address and/or mobile phone number at T0 or T1, they also received an email message and/or a short message service (SMS) text message to remind them to complete the final Web-based questionnaire.

### Measurements

#### Overview

The primary outcome measures were intention to start smoking and the smoking behavior of the participating children. Both measures were assessed at T0, T1, and T2, and were based on self-reports using a previously used staging question [18].

#### Intention to Start Smoking

For intention to start smoking, children could indicate which one of seven statements would describe their intentions best. The statements were the following: “I am sure I will never start smoking,” “I think I will never start smoking,” “I think I will start smoking in the future,” “I think I will start smoking within 5 years,” “I think I will start smoking within 1 year,” “I think I will start smoking within 6 months,” and “I think I will start smoking within 1 month.” Children who indicated that they intended to start smoking anytime in the future were categorized as having the intention to smoke (scored as 1). Children who indicated they would never start smoking in the future (ie, “I am sure I will never start smoking” and “I think I will never start smoking”) were categorized as not having the intention to smoke (scored as 0).

#### Smoking Behavior

Smoking behavior of the children was also assessed by a staging algorithm. Children could indicate which one of nine statements described their smoking behavior best. The statements were the following: “I have never smoked, not even a puff,” “I have tried smoking, but do not smoke anymore,” “I stopped smoking, I used to smoke less than once a month,” “I stopped smoking after I smoked at least once a week,” “I try smoking occasionally,” “I smoke less than once a month,” “I do not smoke every week, but at least once a month,” “I do not smoke daily, but at least once a week,” and “I smoke at least once a day.” Children who indicated that they were smokers (ie, occasionally, monthly, weekly, or daily) were scored with a 1, otherwise a child was considered to be a nonsmoker (ie, never smoked or stopped smoking) and scored with a 0.

#### Background Variables

Background variables measured the age in years, gender (boy=1, girl=2), SES, and ethnicity of the participating children.

##### Socioeconomic Status

SES of the participating children was based on their postal code, which they had provided in the questionnaire. The Netherlands Institute for Social Research—a Dutch government agency that conducts research into the social aspects of all areas of government policy—calculated an index score for the 4-digit postal code of all Dutch inhabitants, based on their income, occupation, and education [34,35]. Based on this index score, children living in a low SES neighborhood were coded with a 0, and children living in a high SES neighborhood were coded with a 1.

##### Ethnicity

To assess ethnicity, children reported their parents’ and their own places of birth. In line with the guidelines of Statistics Netherlands, a child was considered to have a Western ethnic background (coded as 1) if he/she and both parents had been born in the Netherlands, another European country, North America, Oceania, Indonesia (a former Dutch colony), or Japan. Otherwise, the child was considered to have a non-Western ethnic background (coded as 2) [36].

#### Sociocognitive Variables

Sociocognitive variables were only measured at T0 and T1 and were derived from the I-Change Model [37].

##### Attitude

Attitude was measured toward both the positive and negative consequences of smoking and answers were scored on a 4-point scale. Advantageous attitudes (Cronbach alpha=.85) included nine questions concerning the benefits of smoking (eg, feeling mature, cool, or sociable), whereas disadvantageous attitudes (Cronbach alpha=.81) comprised ten questions concerning the drawbacks of smoking (eg, less physically fit, will become ill, or will get addicted).

##### Social Influence

Social influence was measured using perceived social norms (ie, norms about the smoking status of important people in the child’s environment) and modeling (ie, the smoking behavior of people in the child’s environment). The social norm measure included seven questions concerning the norms of the smoking status of the children’s father, mother, brother(s), sister(s), friends, best friend, and most people that are important to them. These questions were scored on a 5-point Likert scale (Cronbach alpha=.70). The modeling measure included eight questions about the smoking behavior of the children’s father, mother, brother(s), sister(s), and best friend. Furthermore, the number of friends, family members, and classmates who smoked was assessed. The modeling measure questions were also scored on a 5-point Likert scale.

##### Self-Efficacy

Self-efficacy expectations were measured with ten questions concerning the ability of the child to refuse cigarettes in different situations. Participants indicated how easy or difficult it was to refuse cigarettes using a 5-point Likert scale, where -2 was “very difficult” and +2 was “very easy” (Cronbach alpha=.94).

### The Fun Without Smokes Intervention

#### Overview

All participating children received personalized log-in codes to access the Fun without Smokes website and to complete the measurements at T0, T1, and T2. The Fun without Smokes intervention consisted of computer-tailored feedback messages (ie, educational content) and prompt messages to stimulate reuse (ie, additional log-ins and views) of the Fun without Smokes website. In the coming paragraphs, the Fun without Smokes intervention will be explained for each study arm.

#### No-Prompt Group

After completion of the Web-based questionnaire, children randomized into the no-prompt group received three computer-tailored feedback messages on three consecutive days. The first feedback message provided advice on the children’s attitude toward smoking, the second provided advice on perceived social influence, and the third addressed children’s self-efficacy expectations concerning their ability to refuse cigarettes. The messages were sent to the children’s email addresses as a PDF file and were also available at the Fun without Smokes website. The computer-tailored feedback messages were tailored to the children’s personal (ie, name, age, and gender) and sociocognitive characteristics (ie, attitude, social influence, and self-efficacy expectations), which they had provided in the Web-based questionnaire.

#### Prompt Group

Similar to the children in the no-prompt group, children randomized to the prompt group received three computer-tailored feedback messages upon completion of the Web-based questionnaire. However, children in the prompt group also received six prompt messages via email and SMS every year encouraging them to reuse the Fun without Smokes website. At the Fun without Smokes website, children were able to read smoking and nonsmoking information, watch animated videos with nonsmoking content, play games concerning nonsmoking, fill out the Web-based questionnaire, or read the computer-tailored feedback messages. The aim of the website was to repeatedly expose children to nonsmoking information during the course of the year in addition to the tailored feedback messages. The content of the website changed regularly to include new information and interactive elements. The prompt messages were sent to announce a new topic related to smoking prevention that was addressed at the Fun without Smokes website (ie, games, animated videos, or new smoking and nonsmoking information). Children in the prompt and no-prompt group were able to reuse the Fun without Smokes website during the entire intervention period. However, children in the no-prompt group were not prompted to reuse the website.

Among the prompt and no-prompt group, significant differences were observed in the numbers of reuse actions of the Fun without Smokes website after the Web-based questionnaire was completed. Between T0 and T1, the mean number of reuse actions was 2.14 (SD 7.53) in the prompt group and 0.47 (SD 2.30) in the no-prompt group [38]. Between T1 and T2, the mean number of reuse actions was 0.67 (SD 2.79) in the prompt group and 0.06 (SD 0.63) in the no-prompt group.

#### Control Group

Children in the control group also completed the Web-based questionnaire at the Fun without Smokes website, but did not receive computer-tailored feedback or prompt messages. They were only able to use the intervention website during the three measurement sessions and not during the intervening periods. They also did not have access to the nonsmoking information or interactive elements of the website. More detailed information about the Fun without Smokes study is available elsewhere [32].

### Statistical Analyses

Children in this study were nested in schools and, therefore, multilevel analyses were performed. Attrition analysis was done using multilevel logistic regression analysis to assess which factors (ie, age, gender, ethnicity, SES, advantageous or disadvantageous attitudes, social norms, modeling, self-efficacy, smoking behavior at T0, and intention to start smoking at T0) could explain the dropout between T0 and T1, and between T0 and T2. To describe the demographic characteristics of the children at T0 and potential differences concerning their primary outcome measures, general descriptive analyses were carried out (ie, means, standard deviations, and percentages) on the children that participated in the baseline measurement. Furthermore, analysis of variance (ANOVA) was used to assess whether attitude, social influence, and self-efficacy expectations differed between the study groups at T0. Additionally, to determine the number of children that changed their smoking intentions or smoking behaviors during the intervention period, basic analyses were performed to report the transition from a negative intention at T0 to a positive intention to engage in smoking at T1 and T2. The transition of nonsmoking at T0 to smoking at T1 and T2 was also analyzed using the data provided by the children that participated in the measurements at T0, T1, and T2.

Multilevel logistic regression analyses were done to assess the program effects on the prompt and no-prompt group as compared to the control group. Separate analyses were performed to assess the intervention effects on smoking intention and smoking behavior among children at T1 and T2. These analyses were adjusted for age, gender, ethnicity, SES, advantageous attitude, disadvantageous attitude, social norms, modeling, and self-efficacy. In the analyses concerning both the smoking behavior and intention to start smoking, children who smoked at T0 were excluded from the analyses. Because of the high dropout rate, multiple imputation of missing variables was applied. In the multiple imputation analyses, missing values were imputed using the intervention factor (ie, control-prompt and control-no-prompt), background variables (ie, age, gender, ethnicity, and SES), smoking intention, smoking behavior, and sociocognitive variables (ie, advantageous or disadvantageous attitudes, social norms, modeling, and self-efficacy) as predictor variables. Based on the percentage of missing data, a total of 50 datasets were imputed for T0 and T1 data, and 62 datasets were imputed for T0 and T2 data [39,40]. The program effects were analyzed by averaging the results of all the datasets (pooling). The prompt and no-prompt group were dummy coded with the control group as a reference. We set about to identify whether there were differential effects between the control, prompt, and no-prompt groups based on children’s smoking intentions or smoking behavior at T1 and T2. To do this, we performed multilevel logistic regression analysis that included SES as the only interaction term to examine potential differences among children living in high- and low-SES neighborhoods. Those analyses were adjusted for age, gender, ethnicity, advantageous and disadvantageous attitude, social norms, modeling, and self-efficacy. If an interaction effect by SES was present, separate analyses were performed for the high- and low-SES groups. All analyses were performed using SPSS version 20.0 and MLwiN version 2.28 [41]. Differences were considered significant when *P*≤.05. Interaction effects were considered to be significant when *P*≤.10.

## Results

### Attrition Analyses

A total of 3213 children participated in the Fun without Smokes study at T0. Between T0 and T1 a total of 1067 children out of the original 3213 (33.21%) dropped out of the study. Between T0 and T2 the number of children that did not participate in the final measurement was 1730 out of the original 3213 (53.84%).

The attrition analysis showed that older children were more likely to drop out at T1 (odds ratio [OR] 1.30, 95% CI 1.01-1.67). At T2, children dropped out more frequently if they were boys (OR 0.64, 95% CI 0.55-0.75), older (OR 1.25, 95% CI 1.07-1.46), had a non-Western ethnic background (OR 1.51, 95% CI 1.17-1.94), were randomized into the prompt group as compared to the control group (OR 1.43, 95% CI 1.16-1.78), and had more smokers in their environment (OR 1.73, 95% CI 1.37-2.19).

### Sample Characteristics

Table 1 shows the characteristics of the children at T0 that were randomized into the prompt, no-prompt, and control groups. Overall, slightly more girls (1625/3213, 50.58%) participated in the first measurement and the majority of the children had a Western ethnic background (2836/3213, 88.27%). At T0, 3.39% (109/3213) had a positive intention to start smoking and 1.15% (37/3213) indicated current smoking behavior. In the control group, significantly more children (*P*<.001) were of high SES, as compared to the prompt and no-prompt groups. No significant differences (*P*>.05) between study groups at T0 were observed for the other smoking-related factors (ie, attitude, social influence, and self-efficacy expectations).

Table 2 indicates that between T0 and T1, 48 children out of 2006 (2.39%) changed their negative intention to engage in smoking into a positive one. Additionally, a total of 9 children out of 2094 (0.43%) actually started to smoke after 12 months of follow-up. After 25 months, 23 children out of 1402 (1.64%) indicated they had a positive intention to start smoking and 13 children out of 1462 (0.89%) indicated current smoking behavior.

**Table 1:** Sample characteristics of children in each study group at T0.

Sample characteristic	Study group						
	Overall (n=3213)	Prompt (n=1207)	No-prompt (n=1003)	Control (n=1003)	*F* test (df)	*P* value	Missing values, n (%)
Age in years, mean (SD)	10.36 (0.55)	10.36 (0.55)	10.35 (0.54)	10.38 (0.55)	1.02 (2)	.36	107 (3.33)
Gender (female), n (%)	1625 (50.59)	618 (51.20)	495 (49.35)	512 (51.05)	0.44 (2)	.65	0 (0)
SES (high), n (%)	1354 (42.14)	440 (36.45)	431 (42.97)	483 (48.16)	13.21 (2)	<.001^a^	577 (17.96)
Ethnicity (Western), n (%)	2836 (88.27)	1072 (88.82)	875 (87.23)	889 (88.63)	0.75 (2)	.48	9 (0.28)
Intention to smoke at T0 (positive), n (%)	109 (3.39)	35 (2.90)	37 (3.69)	37 (3.69)	0.76 (2)	.47	75 (2.33)
Smoking behavior at T0 (smoker), n (%)	37 (1.15)	16 (1.33)	10 (1.00)	11 (1.10)	0.26 (2)	.77	34 (1.06)

^a^Significantly more children in the control group were of high SES, as compared to the prompt and no-prompt groups.

**Table 2:** Transition of intention to start smoking and smoking behavior between T0 and T1, and between T0 and T2.

Transition of smoking intention and behavior between measurement sessions		Study group				
		Overall	Prompt	No-prompt	Control	Missing values, n (%)
**T0 to T1**						
	Negative to positive smoking intention, n (%)	48/2006 (2.39)	13/701 (1.85)	18/623 (2.89)	17/682 (2.49)	1023/3029 (33.77)
	Nonsmoking to smoking behavior, n (%)	9/2094 (0.43)	4/727 (0.55)	2/649 (0.31)	3/718 (0.42)	1048/3142 (33.35)
**T0 to T2**						
	Negative to positive smoking intention, n (%)	23/1402 (1.64)	7/491 (1.43)	10/446 (2.24)	6/465 (1.29)	1627/3029 (53.71)
	Nonsmoking to smoking behavior, n (%)	13/1462 (0.89)	3/504 (0.59)	5/470 (1.06)	5/488 (1.02)	1680/3142 (53.47)

### Intervention Effects on Smoking Intention and Smoking Behavior

The program effects on the intention to start smoking between T0 and T1, and between T0 and T2, were calculated. Multilevel logistic regression analyses indicated no significant differences at T1 between the control and prompt groups (OR 0.67, 95% CI 0.30-1.50) or between the control and no-prompt groups (OR 0.76, 95% CI 0.34-1.67). Similar nonsignificant effects concerning the intention to start smoking were observed at T2 between the control and prompt groups (OR 0.78, 95% CI 0.26-2.32) and between the control and no-prompt groups (OR 1.31, 95% CI 0.45-3.82).

The program effects on smoking behavior between T0 and T1, and between T0 and T2, were calculated. Multilevel logistic regression analyses indicated that at T1, no significant program effects were found between the control and prompt groups (OR 1.13, 95% CI 0.13-9.98). As well, no significant program effects were found between the control and no-prompt groups (OR 0.50, 95% CI 0.04-5.59). At T2, no significant differences in smoking behavior were observed between the control and prompt groups (OR 0.53, 95% CI 0.12-2.47) or between the control and no-prompt groups (OR 1.01, 95% CI 0.24-4.21).

### Effect of Socioeconomic Status Within Study Groups

The results of the intervention by SES interaction showed that SES did not moderate the association between intention to start smoking and smoking behavior on the one hand and type of intervention at the other, not at T1 nor at T2 (*P*>.10). For that reason, no further subgroup analyses were performed.

## Discussion

### Principal Findings

The main aim of this study was to evaluate whether computer-tailored feedback messages, with and without prompt messages, are effective in decreasing smoking intention and smoking behavior of Dutch primary school children (aged 10 to 12 years) after 12 and 25 months of follow-up. Feedback messages were meant to stimulate reuse of the intervention website and increase exposure to the nonsmoking information. Since the smoking initiation and smoking intention rates were low among the study sample, findings of this study indicate that the two versions of the intervention—prompt and no-prompt messages—were not able to reduce smoking intention and smoking behavior at either of the time points.

The findings of this study are in line with the results reported by a recently published, home-based smoking prevention program [42] in which no preventive effects were found in smoking initiation among Dutch primary school children. In that study, the nonsignificant findings were attributed to the low smoking prevalence rates at this young age. This explanation is also valid for our study since the smoking intention and smoking initiation rates were low in all study arms, ranging from 1.29% to 2.89% and from 0.31% to 1.06%, respectively. The Fun without Smokes study was based on the out-of-school intervention of the Octopus study which was shown to be effective in preventing the initiation and continuation of smoking among primary school children (aged 11 to 12 years) [18]. However, the Octopus study started over a decade ago when monthly smoking prevalence rates among children were higher (2% to 5% smoking prevalence at 10 to 12 years of age), whereas nowadays those prevalence rates are 0% [7]. This decreasing trend may have been caused due to the implementation of policies to reduce smoking (ie, smoking bans in public places and workplaces, or tax increases) [43,44] or the changing norms regarding smoking [45]. Thereby, the smoking initiation rates have shifted to later ages among children and adolescents during the last ten years [7]. Hence, a smoking prevention program for 10 to 12 year olds may not be as relevant as it would be for adolescents attending secondary school when the actual uptake of smoking starts [6,7]. This suggestion has also been supported by other studies [16,20] that reported positive preventive effects for smoking prevention interventions among adolescents.

Besides the societal and smoking-climate changes during the last decade, other factors might have contributed to the nonsignificant findings of the Fun without Smokes study and should be considered in the development of future Web-based interventions. The Octopus study was a paper-based program, whereas the Fun without Smokes study was delivered via Internet. Although previous research already showed that Web-based interventions were effective in decreasing substance use [46] and improving dietary behavior [47] among children, no consistent evidence for effective Web-based smoking prevention interventions has been found yet. The educational content of both the Octopus study and the Fun without Smokes study was provided via computer-tailored feedback messages, but the content and extensiveness of the information provided was not the same. Tailored information via the Internet should be concise [48] and, therefore, the messages were edited down to brief messages that were suitable for provision through the Internet. A further dissimilarity between the studies was the difference in follow-up measurements. Both studies used multiple measurements and included both the transition periods from grade 7 to grade 8, and from primary to secondary school. In the Octopus study, there were follow-up measurements at 6, 9, 20, 30, and 36 months and in the Fun without Smokes study after 12 and 25 months. However, positive effects were observed in the Octopus study only at 6 months of follow-up [18]. This may indicate that the positive preventive effects in the Fun without Smokes study were missed because follow-up periods were too long. Moreover, in the Octopus study and the Fun without Smokes study, a similar staging question was used to assess children’s smoking behavior, but the studies categorized smokers and nonsmokers differently—the Octopus study used the categories *never smokers*, *noncurrent smokers*, and *current smokers*, while the Fun without Smokes study used the categories *smokers* and *nonsmokers*. It is, however, unlikely that this difference explains the nonsignificant findings in the Fun without Smokes study, since the smoking prevalence rates of the children were low.

Despite the nonsignificant findings in the present study, it has been recommended that smoking prevention programs start before attitudes and beliefs toward smoking are formed [8], since the transition period of primary to secondary school is a crucial time when adolescents may engage in smoking [49]. Therefore, it is to be expected that smoking prevention programs may influence sociocognitive factors (ie, attitude, social influence, and self-efficacy expectations) even if no effects on smoking intentions and smoking behavior are indicated. A review by Hopfer et al [50] revealed that substance use (ie, alcohol, cigarette smoking, and other drugs) prevention programs were able to change children’s attitudes, subjective norms, self-efficacy expectations, and knowledge. Similar results are observed among other studies [22,51] in which Web-based interventions demonstrated changes in behavioral determinants. However, at the final measurement session of this study, no sociocognitive factors were assessed. Therefore, no statements can be made about whether the computer-tailored feedback messages or the prompt messages were able to change sociocognitive factors.

To increase the likelihood of lower smoking intention and smoking behavior rates, this study used prompt messages to stimulate reuse of the intervention website. Although a prior study [38] showed that prompt messages were able to stimulate reuse of the Fun without Smokes website, the percentage of reuse remained low and, therefore, the exposure to the nonsmoking content was limited. This may have been caused by the content of the prompt messages. The content may not have been stimulating enough, or the children may have felt they had no reason to reuse the Fun without Smokes website. Another reason may have been the timing or frequency of the prompt messages. The first three prompt messages were sent at one-month time intervals, whereas the last three prompts were sent every two months. Previous studies [52,53] indicated that shorter time between prompts would be most effective. However, no unequivocal conclusion has been reported for the optimal prompt timing and frequency. Additionally, national reports [12,54,55] indicated that the majority of Dutch primary school children have an email address and mobile phone, though the actual use of those devices among this age group may be low. Therefore, future research should not only focus on optimal prompt timing, frequency, and content, but also on effective delivery channels to stimulate children to reuse an intervention website.

### Strengths and Limitations

A strength of this study is the large sample of Dutch primary school children, since children from all regions in the Netherlands participated in the Fun without Smokes study. Another strength is the follow-up period of 25 months, which enables a long-term evaluation of the intervention effects. However, this study was also subject to some limitations. Since a large number of schools were approached for participation (n=3500) and only 162 schools were able to participate, the study sample should not be seen as representative of all schools in the Netherlands and outside the Netherlands. However, the smoking prevalence rates that were found in this study are comparable to those found in Dutch national reports [6], indicating that there were no large differences between our sample and the Dutch population of primary school children in terms of smoking prevalence. Due to differences in smoking prevalence between the Netherlands and other countries, the results may be less generalizable to countries with higher smoking prevalence rates among children aged 10 to 12 years. Another limitation may be the lack of a process evaluation of the Fun without Smokes intervention. Therefore, we did not receive in-depth information concerning the children’s opinions toward the intervention, which may explain the nonsignificant differences among the three study arms. However, children had to complete the final questionnaire at home on their own initiative. To increase the likelihood that children answered all of the questions, we chose to leave out a process evaluation. Nevertheless, it is advisable for future research to evaluate the process of the Web-based intervention, which may affect the direction of further research. A final limitation is the use of the SES index score that was based on the children’s postal code. This index score reflects the SES at a neighborhood level and not the individual SES of children. The SES measure that was used was based on the income, occupation, and education of the inhabitants living in that neighborhood and is known to correlate strongly with a more precise 6-digit postal code [35].

### Conclusions

No program effects in the intervention groups (ie, prompt and no-prompt groups) were found in the Web-based, computer-tailored smoking prevention trial at 12 and 25 months of follow-up. This indicates that the Web-based, computer-tailored feedback messages were not able to change the smoking intentions and smoking behaviors of the participating children. Although the prompt messages were meant to stimulate reuse of the intervention website, resulting in an increased exposure to the nonsmoking information, no effects were observed concerning the smoking intentions and smoking behaviors of children in the prompt group. It is not completely clear why the Fun without Smokes intervention was not found to be effective. This may have been caused by the low smoking initiation rates among children and the lack of exposure to the intervention content. Future Web-based smoking prevention programs should, therefore, take place closer to the age of actual smoking uptake (ie, secondary school). Furthermore, future evaluations of smoking prevention interventions should focus on assessing and controlling exposure to the intervention content and the response to the prompt messages.

## Multimedia Appendix 1

CONSORT-EHEALTH checklist V1.6.2 [56].

## Abbreviations

ANOVA: analysis of variance
OR: odds ratio
SES: socioeconomic status
SMS: short message service
