# Supplementary material for: A Web-Based, Computer-Tailored Smoking Prevention Program to Prevent Children From Starting to Smoke After Transferring to Secondary School: Randomized Controlled Trial
Source: J Med Internet Res. 2015 Mar 9;17(3):e59. doi: 10.2196/jmir.3794 (PMC4371791; doi:10.2196/jmir.3794)
Supplement: Supplementary file 1 [file jmir_v17i3e59_app1.pdf]

CONSORT-EHEALTH Checklist V1.6.2 Report

(based on CONSORT-EHEALTH V1.6), available at [http://tinyurl.com/consort-ehealth-v1-6].

3794

Date completed

8/18/2014 5:01:37

by

Henricus-Paul Cremers

Is a Web-based Computer-tailored Smoking Prevention Program effective in Preventing Children to start Smoking after they transfer to Secondary School? Findings of a Randomized Controlled Trial at 12 and 25 Months of Follow-up

TITLE

1a-i) Identify the mode of delivery in the title

"web-based"

1a-ii) Non-web-based components or important co-interventions in title

1a-iii) Primary condition or target group in the title

"preventing children to start smoking after they transfer to secondary school"

ABSTRACT

1b-i) Key features/functionalities/components of the intervention and comparator in the METHODS section of the ABSTRACT

"A total of 3213 children (10 – 12 years) participated in the study and completed a web-based questionnaire assessing their smoking intention, smoking behavior and socio-cognitive factors (i.e. attitude, social influence and self-efficacy) related to smoking. After completion, children in the intervention groups received computer-tailored feedback letters that were sent by email and that could be accessed on the intervention website. Children in the prompt group received prompt messages (via email and SMS) to stimulate them to reuse the intervention website with non-smoking content."

1b-ii) Level of human involvement in the METHODS section of the ABSTRACT

1b-iii) Open vs. closed, web-based (self-assessment) vs. face-to-face assessments in the METHODS section of the ABSTRACT

1b-iv) RESULTS section in abstract must contain use data

1b-v) CONCLUSIONS/DISCUSSION in abstract for negative trials

INTRODUCTION

2a-i) Problem and the type of system/solution

"Smoking among children and adolescents remains a public health problem [1-3], potentially leading to chronic diseases, cardiovascular diseases or cancer at a later age [4, 5]. Although, smoking prevalence among Dutch primary school children at the age of 12 has decreased in the past decade from 2 – 5% to 0% [6, 7] this percentage still increases rapidly when children are in secondary school (13% is a daily smoker at age 15) [6]. One prevention strategy suggested is to start smoking prevention programs already at primary school, before positive beliefs toward smoking are formed [8]. Given the advantages of a web-based computer-tailored approach (i.e. reduced cost and an expanded reach of participants) [9-11] and the increasing use of the Internet among Dutch children (i.e. 96% Internet use at 11 – 14 years of age) [12] web-based computer-tailored smoking prevention programs may be helpful in decreasing smoking initiation rates among children."

2a-ii) Scientific background, rationale: What is known about the (type of) system

"Previous studies [9, 10, 19] reported web-based computer-tailored programs to be effective in changing unhealthy behaviors both among adults, adolescents and children."

"This requires the use of optimal strategies to improve adherence. According to previous research [25-29] prompt messages may be effective in stimulating participants to reuse a web-based intervention. However, using prompt messages in smoking prevention trials has not been studied among children before."

METHODS

3a) CONSORT: Description of trial design (such as parallel, factorial) including allocation ratio

"The aim of this study was to evaluate whether computer-tailored feedback letters with and without prompt messages are effective in decreasing the smoking intentions and smoking behavior of Dutch primary school children (aged 10 – 12 years) after 12 and 25 months of follow-up. Furthermore, it is known that children with a low socioeconomic status (SES) engage more often in smoking [30] and have a higher intention to start smoking [31], as compared to high SES children. Therefore it will also be assessed whether SES moderates the effects of the two versions of the intervention."

3b) CONSORT: Important changes to methods after trial commencement (such as eligibility criteria), with reasons

Not applicable

3b-i) Bug fixes, Downtimes, Content Changes

4a) CONSORT: Eligibility criteria for participants

"Children of all participating schools were all included in the intervention trial at T0, unless they or their parents refused to be involved (1.7% refused at T0). The participating children in the present study were Dutch primary school children of grade 7 (aged 10 – 11 years), they were followed in grade 8 (T1) and when they transferred to secondary school (T2)."

4a-i) Computer / Internet literacy

4a-ii) Open vs. closed, web-based vs. face-to-face assessments:

"For the present study approximately 3500 primary schools were approached by seven Dutch Municipal Health Promotion Organizations and Maastricht University."

4a-iii) Information giving during recruitment

4b) CONSORT: Settings and locations where the data were collected

"At T0 and T1 children completed the web-based questionnaire at their primary school under supervision of their teacher. At T2 children made the transition to secondary school and had to complete the web-based questionnaire outside school on their own initiative. In this period all children who participated at T0 received an information letter sent by postal mail at their home address to complete the web-based questionnaire for the last time. If children had provided their email address and/or mobile phone number at T0 or T1, they also received email and/or short message service (SMS) messages to remind them to complete the final web-based questionnaire."

4b-i) Report if outcomes were (self-)assessed through online questionnaires

"Both measures were assessed at T0, T1 and T2 and based on self-reports using a previously used staging question [18]."

4b-ii) Report how institutional affiliations are displayed

5) CONSORT: Describe the interventions for each group with sufficient details to allow replication, including how and when they were actually administered

5-i) Mention names, credential, affiliations of the developers, sponsors, and owners

5-ii) Describe the history/development process

5-iii) Revisions and updating

5-iv) Quality assurance methods

5-v) Ensure replicability by publishing the source code, and/or providing screenshots/screen-capture video, and/or providing flowcharts of the algorithms used

5-vi) Digital preservation

5-vii) Access

"At T0 and T1 children completed the web-based questionnaire at their primary school under supervision of their teacher. At T2 children made the transition to secondary school and had to complete the web-based questionnaire outside school on their own initiative. In this period all children who participated at T0 received an information letter sent by postal mail at their home address to complete the web-based questionnaire for the last time. If children had provided their email address and/or mobile phone number at T0 or T1, they also received email and/or short message service (SMS) messages to remind them to complete the final web-based questionnaire."

5-viii) Mode of delivery, features/functionalities/components of the intervention and comparator, and the theoretical framework

"Children randomized to the no prompt group received after completion of the web-based questionnaire, three computer-tailored feedback letters on three consecutive days. The first feedback letter provided advice on children's attitude toward smoking, the second on the perceived social influence and the third addressed children's self-efficacy expectations concerning refusing cigarettes. The letters were sent to the email address (as a PDF file) and were also available at the "Fun without Smokes" website. Those computer-tailored feedback letters were tailored to children's personal characteristics (i.e. name, age and gender) and to their socio-cognitive factors (i.e. attitude, social influence and self-efficacy expectations), which they had provided in the web-based questionnaire."

"Similar to the children in the no prompt group, children randomized to the prompt group received three computer-tailored feedback letters after completion of the web-based questionnaire. However, children in the prompt group also received six prompt messages (via email and SMS) every year to stimulate them to reuse the "Fun without Smokes" website."

5-ix) Describe use parameters

5-x) Clarify the level of human involvement

5-xi) Report any prompts/reminders used

"Similar to the children in the no prompt group, children randomized to the prompt group received three computer-tailored feedback letters after completion of the web-based questionnaire. However, children in the prompt group also received six prompt messages (via email and SMS) every year to stimulate them to reuse the "Fun without Smokes" website."

5-xii) Describe any co-interventions (incl. training/support)

Not applicable

6a) CONSORT: Completely defined pre-specified primary and secondary outcome measures, including how and when they were assessed

"The primary outcome measures were intention to start smoking and the smoking behavior of the participating children. Both measures were assessed at T0, T1 and T2 and based on self-reports using a previously used staging question [18]."

"Socio-cognitive variables were only measured at T0 and T1 and were derived from the I-Change Model [37]."

6a-i) Online questionnaires: describe if they were validated for online use and apply CHERRIES items to describe how the questionnaires were designed/deployed

6a-ii) Describe whether and how "use" (including intensity of use/dosage) was defined/measured/monitored

6a-iii) Describe whether, how, and when qualitative feedback from participants was obtained

6b) CONSORT: Any changes to trial outcomes after the trial commenced, with reasons

Not applicable

7a) CONSORT: How sample size was determined

7a-i) Describe whether and how expected attrition was taken into account when calculating the sample size

7b) CONSORT: When applicable, explanation of any interim analyses and stopping guidelines

Not applicable

8a) CONSORT: Method used to generate the random allocation sequence

"Primary schools were the unit of randomization and were randomly assigned to one of the three study arms (i.e. prompt, no prompt or control group) in a computer determined sequence using a clustered randomization scheme."

8b) CONSORT: Type of randomisation; details of any restriction (such as blocking and block size)

"cluster-randomized controlled trial"

9) CONSORT: Mechanism used to implement the random allocation sequence (such as sequentially numbered containers), describing any steps taken to conceal the sequence until interventions were assigned

Not applicable

10) CONSORT: Who generated the random allocation sequence, who enrolled participants, and who assigned participants to interventions

"Primary schools were the unit of randomization and were randomly assigned to one of the three study arms (i.e. prompt, no prompt or control group) in a computer determined sequence using a clustered randomization scheme."

11a) CONSORT: Blinding - If done, who was blinded after assignment to interventions (for example, participants, care providers, those assessing outcomes) and how

11a-i) Specify who was blinded, and who wasn't

Not applicable

11a-ii) Discuss e.g., whether participants knew which intervention was the "intervention of interest" and which one was the "comparator"

11b) CONSORT: If relevant, description of the similarity of interventions

"Children randomized to the no prompt group received after completion of the web-based questionnaire, three computer-tailored feedback letters on three consecutive days. The first feedback letter provided advice on children's attitude toward smoking, the second on the perceived social influence and the third addressed children's self-efficacy expectations concerning refusing cigarettes. The letters were sent to the email address (as a PDF file) and were also available at the "Fun without Smokes" website. Those computer-tailored feedback letters were tailored to children's personal characteristics (i.e. name, age and gender) and to their socio-cognitive factors (i.e. attitude, social influence and self-efficacy expectations), which they had provided in the web-based questionnaire."

"Similar to the children in the no prompt group, children randomized to the prompt group received three computer-tailored feedback letters after completion of the web-based questionnaire. However, children in the prompt group also received six prompt messages (via email and SMS) every year to stimulate them to reuse the "Fun without Smokes" website. At the "Fun without Smokes" website children were able to read (non-)smoking information, watch animated videos with non-smoking content, play games concerning non-smoking, fill-out the web-based questionnaire or read the computer-tailored feedback letters. The aim of the website was to repeatedly expose children to non-smoking information during the course of the year in addition to the tailored feedback letters. The content of the website changed regularly to include new information and interactive elements and the prompt messages were sent to announce a new topic related to smoking prevention that was addressed at the "Fun without Smokes" website (i.e. games, animated videos or new (non-)smoking information). Children in the prompt and no prompt group were able to reuse the "Fun without Smokes" website during the entire intervention period. However, children in the no prompt group were not prompted to reuse the website."

12a) CONSORT: Statistical methods used to compare groups for primary and secondary outcomes

"Children in the present study were nested in schools and therefore multilevel analyses were performed. Attrition analysis was done using multilevel logistic regression analysis to assess which factors (i.e. age, gender, ethnicity, SES, attitude (advantages), attitude (disadvantages), social norm, modeling, self-efficacy, smoking behavior at T0 and intention to start smoking at T0) could explain the drop-out between T0 and T1 and between T0 and T2. To describe the demographic characteristics of the children at T0 and potential differences concerning their primary outcome measures, general descriptive analyses were carried out (i.e. means, standard deviations and percentages) on the children that participated in the baseline measurement. Furthermore, analysis of variance (ANOVA) was used to assess if attitude, social influence and self-efficacy expectations differed between the study groups at T0. Additionally, to indicate the number of children that changed their smoking intention or smoking behavior during the intervention period basic analyses were performed to report the transition from a negative intention at T0 to a positive intention to engage in smoking at T1 and T2. The transition of non-smoking at T0 to smoking at T1 and T2 was also analyzed, using the data from the children that participated in the measurements at T0, T1 and T2.

Multilevel logistic regression analyses were done to assess the program effects in the prompt and no prompt group as compared to the control group. Separate analyses were performed to assess the intervention effects on smoking intention and smoking behavior among children at T1 and T2. These analyses were adjusted for age, gender, ethnicity, SES, attitude (advantages), attitude (disadvantages), social norm, modeling and self-efficacy. In the analyses concerning the smoking behavior and intention to start smoking children who smoked at T0 and children who had the intention to start smoking at T0 were excluded from the study sample. Because of the high drop-out multiple imputation of missing variables was applied. In the multiple imputation analyses missing values were imputed using the intervention factor (i.e. control-prompt and control-no prompt), background variables (i.e. age, gender, ethnicity and SES), smoking intention, smoking behavior and the socio-cognitive variables (i.e. attitude (advantages and disadvantages), social influence (social norm and modeling) and self-efficacy) as predictor variables. Based on the percentage of missing data, a total of 50 datasets were imputed for the data of T0 and T1, and 62 datasets were imputed for the data of T0 and T2 [38, 39]. The program effects were analyzed by averaging the results of all the datasets (pooling). The prompt and no prompt group were dummy coded with the control group as a reference. To identify whether there were differential effects between the control, prompt and no prompt group based on children's smoking intentions or smoking behavior at T1 and T2, a multilevel logistic regression analysis was performed that included only SES as interaction term to examine potential differences among children living in high and low SES neighborhoods. Those analyses were adjusted for age, gender, ethnicity, attitude (advantages), attitude (disadvantages), social norm, modeling and self-efficacy. If an interaction effect of SES was present, separate analyses were performed for the high and low SES groups. All analyses were performed in SPSS 20.0 and MLwiN 2.28 [40]. P-values were found to be significant if they were equal to or lower than .05. Interaction effects were considered to be significant if the P-value was equal to or lower than .10."

12a-i) Imputation techniques to deal with attrition / missing values

"Because of the high drop-out multiple imputation of missing variables was applied. In the multiple imputation analyses missing values were imputed using the intervention factor (i.e. control-prompt and control-no prompt), background variables (i.e. age, gender, ethnicity and SES), smoking intention, smoking behavior and the socio-cognitive variables (i.e. attitude (advantages and disadvantages), social influence (social norm and modeling) and self-efficacy) as predictor variables. Based on the percentage of missing data, a total of 50 datasets were imputed for the data of T0 and T1, and 62 datasets were imputed for the data of T0 and T2 [38, 39]. The program effects were analyzed by averaging the results of all the datasets (pooling). The prompt and no prompt group were dummy coded with the control group as a reference."

12b) CONSORT: Methods for additional analyses, such as subgroup analyses and adjusted analyses

"To identify whether there were differential effects between the control, prompt and no prompt group based on children's smoking intentions or smoking behavior at T1 and T2, a multilevel logistic regression analysis was performed that included only SES as interaction term to examine potential differences among children living in high and low SES neighborhoods. Those analyses were adjusted for age, gender, ethnicity, attitude (advantages), attitude (disadvantages), social norm, modeling and self-efficacy. If an interaction effect of SES was present, separate analyses were performed for the high and low SES groups."

RESULTS

13a) CONSORT: For each group, the numbers of participants who were randomly assigned, received intended treatment, and were analysed for the primary outcome

"A total of 3213 children participated in the "Fun without Smokes" study at T0. Between T0 and T1 a total of 1067 children (33.2%) dropped out from the "Fun without Smokes" study, between T0 and T2 the number of children that did not participate in the final measurement was 1730 (53.8%)."

13b) CONSORT: For each group, losses and exclusions after randomisation, together with reasons

"A total of 3213 children participated in the "Fun without Smokes" study at T0. Between T0 and T1 a total of 1067 children (33.2%) dropped out from the "Fun without Smokes" study, between T0 and T2 the number of children that did not participate in the final measurement was 1730 (53.8%).

The attrition analysis showed that children were more likely to drop out at T1 when they had an older age (OR= 1.30; 95%CI= 1.01 – 1.67). At T2 children dropped out more frequently if they were a boy (OR= 0.64; 95%CI= 0.55 – 0.75), had an older age (OR= 1.25; 95%CI= 1.07 – 1.46), had a non-Western ethnic background (OR= 1.51; 95%CI= 1.17 – 1.94), were randomized to the prompt group (as compared to the control group; OR= 1.43; 95%CI= 1.16 – 1.78) and had more smokers in their environment (OR= 1.73; 95%CI= 1.37 – 2.19)."

13b-i) Attrition diagram

14a) CONSORT: Dates defining the periods of recruitment and follow-up

"Children were followed for two years. During this period, children participated at three measurements: baseline (T0= October – November 2011), 12 months of follow-up (T1= October – November 2012) and 25 months of follow-up (T2= November – December 2013)."

14a-i) Indicate if critical "secular events" fell into the study period

14b) CONSORT: Why the trial ended or was stopped (early)

Not applicable, trail was stopped as intended.

15) CONSORT: A table showing baseline demographic and clinical characteristics for each group

"Table 1 shows the characteristics of the children at T0 randomized to the prompt, no prompt and control group. Overall slightly more girls (50.6% (1625/3213)) participated in the first measurement and the majority of the children had a Western ethnic background (88.3% (2836/3213)). At T0 3.4% (109/3213) had a positive intention to start smoking and 1.2% (37/3213) indicated to smoke. In the control group significantly more children (P< .001) were of high SES, as compared to the prompt and no prompt group. No significant differences (P> .05) were observed for the other smoking related factors (i.e. attitude, social influence and self-efficacy expectations) in the study groups at T0."

15-i) Report demographics associated with digital divide issues

Not applicable

16a) CONSORT: For each group, number of participants (denominator) included in each analysis and whether the analysis was by original assigned groups

16-i) Report multiple "denominators" and provide definitions

"Table 1 shows the characteristics of the children at T0 randomized to the prompt, no prompt and control group. Overall slightly more girls (50.6% (1625/3213)) participated in the first measurement and the majority of the children had a Western ethnic background (88.3% (2836/3213)). At T0 3.4% (109/3213) had a positive intention to start smoking and 1.2% (37/3213) indicated to smoke. In the control group significantly more children (P< .001) were of high SES, as compared to the prompt and no prompt group. No significant differences (P> .05) were observed for the other smoking related factors (i.e. attitude, social influence and self-efficacy expectations) in the study groups at T0."

16-ii) Primary analysis should be intent-to-treat

17a) CONSORT: For each primary and secondary outcome, results for each group, and the estimated effect size and its precision (such as 95% confidence interval)

"In table 3 the program effects are shown concerning the intention to start smoking between T0 and T1 and between T0 and T2. Multilevel logistic regression analyses indicated no significant differences between the control and prompt group (OR= 1.05; 95%CI= 0.43 – 2.59) and between the control and no prompt group (OR= 0.80; 95%CI= 0.31 – 2.10) at T1. Similar non-significant effects concerning the intention to start smoking were observed at T2 (control-prompt: OR= 1.20; 95%CI= 0.37 – 3.88); control-no prompt: OR= 1.86; 95%CI= 0.57 – 6.03)."

"The results of the analyses concerning the smoking behavior of the participating children are shown in table 4. At T1 no significant program effects were found between the control and prompt group (OR= 1.13; 95%CI= 0.13 – 9.93) and also not between the control and no prompt group (OR= 0.50; 95%CI= 0.04 – 5.64). Also at T2 no significant differences in smoking behavior were observed between the intervention groups and the control group (control-prompt: OR= 0.53; 95%CI= 0.12 – 2.46); control-no prompt: OR= 1.01; 95%CI= 0.24 – 4.22)."

17a-i) Presentation of process outcomes such as metrics of use and intensity of use

17b) CONSORT: For binary outcomes, presentation of both absolute and relative effect sizes is recommended

Not applicable

18) CONSORT: Results of any other analyses performed, including subgroup analyses and adjusted analyses, distinguishing pre-specified from exploratory

"The results of the intervention by SES interaction showed that SES did not moderate the association between intention to start smoking and smoking behavior on the one hand and type of intervention at the other, not at T1 nor at T2 (P> .10). For that reason no further subgroup analyses were performed."

18-i) Subgroup analysis of comparing only users

19) CONSORT: All important harms or unintended effects in each group

Not applicable

19-i) Include privacy breaches, technical problems

19-ii) Include qualitative feedback from participants or observations from staff/researchers

DISCUSSION

20) CONSORT: Trial limitations, addressing sources of potential bias, imprecision, multiplicity of analyses

20-i) Typical limitations in ehealth trials

"Since a large number of schools were approached for participation (n= 3500) and only 162 schools were able to participate, the study sample should be seen not representative for all schools in the Netherlands and outside the Netherlands. However, the smoking prevalence rates that were found in this study are comparable to those found in Dutch national reports [6], indicating that there were no large differences between our sample and the Dutch population of primary school children in terms of smoking prevalence. Due to differences in smoking prevalence the results may be less generalizable to countries with higher smoking prevalence rates among children (aged 10 – 12 years). Another limitation may be the lack of a process evaluation of the "Fun without Smokes" intervention. Therefore we did not receive in-depth information concerning children's opinions toward the intervention which may explain the non-significant differences among the three study arms. However, children had to complete the final questionnaire at home on their own initiative. To increase the likelihood that children filled-out the number of questions we chose to leave out a process evaluation. Nevertheless, it is advisable for future research to evaluate the process of the web-based intervention which may result in directions for further research. A final limitation is the use of the SES index score that was based on children's postal code. This index score displays the SES at a neighborhood level and not the individual SES of children. The used SES measure was based on the income, occupation and education of the inhabitants living in that neighborhood and is known to correlate high with a more precise 6-digit postal code [35]."

21) CONSORT: Generalisability (external validity, applicability) of the trial findings

21-i) Generalizability to other populations

21-ii) Discuss if there were elements in the RCT that would be different in a routine application setting

22) CONSORT: Interpretation consistent with results, balancing benefits and harms, and considering other relevant evidence

22-i) Restate study questions and summarize the answers suggested by the data, starting with primary outcomes and process outcomes (use)

"The main aim of the present study was to evaluate whether computer-tailored feedback letters with and without prompt messages, to stimulate reuse of the intervention website and increase exposure to the non-smoking information, are effective in decreasing smoking intention and smoking behavior of Dutch primary school children (aged 10 – 12 years) after 12 and 25 months of follow-up. Since the smoking initiation and smoking intention rates were low among the study sample, findings of this study indicate that the two versions of the intervention were not able to reduce smoking intention and smoking behavior at any of the time points."

22-ii) Highlight unanswered new questions, suggest future research

Other information

23) CONSORT: Registration number and name of trial registry
"Netherlands Trial Register Number: NTR3116; http://www.trialregister.nl/ trialreg/admin/rctview.asp?TC=3116 (Archived by WebCite at http://www.webcitation.org /6O0wQYuPI)."

"The "Fun without Smokes" study was approved by the Medical Ethics Committee of the Atrium-Orbis-Zuyd Hospital (NL32093.096.11 / MEC 11-T-25) and registered in the Dutch Trial Register (NTR3116)."

24) CONSORT: Where the full trial protocol can be accessed, if available
"Cremers HP, Mercken L, Oenema A, de Vries H. A web-based computer-tailored smoking prevention programme for primary school children: intervention design and study protocol. BMC Public Health 2012;12:277. doi: 10.1186/1471-2458-12-277 [PMID:22490110]"

25) CONSORT: Sources of funding and other support (such as supply of drugs), role of funders

"This work was supported by ZonMw, the Netherlands Organization for Health Research and Development [200110011]."

X26-i) Comment on ethics committee approval

x26-ii) Outline informed consent procedures

X26-iii) Safety and security procedures

X27-i) State the relation of the study team towards the system being evaluated
